# The Drivers of Pathology in Zoonotic Avian Influenza: The Interplay Between Host and Pathogen

**DOI:** 10.3389/fimmu.2018.01812

**Published:** 2018-08-08

**Authors:** William S. J. Horman, Thi H. O. Nguyen, Katherine Kedzierska, Andrew G. D. Bean, Daniel S. Layton

**Affiliations:** ^1^Department of Microbiology and Immunology, University of Melbourne at the Peter Doherty Institute for Infection and Immunity, Parkville, VIC, Australia; ^2^Australian Animal Health Laboratory, Health and Biosecurity, Commonwealth Scientific and Industrial Research Organisation (CSIRO), East Geelong, VIC, Australia

**Keywords:** avian influenza virus, zoonosis, H7N9, H5N1, highly pathogenic avian influenza virus

## Abstract

The emergence of zoonotic strains of avian influenza (AI) that cause high rates of mortality in people has caused significant global concern, with a looming threat that one of these strains may develop sustained human-to-human transmission and cause a pandemic outbreak. Most notable of these viral strains are the H5N1 highly pathogenic AI and the H7N9 low pathogenicity AI viruses, both of which have mortality rates above 30%. Understanding of their mechanisms of infection and pathobiology is key to our preparation for these and future viral strains of high consequence. AI viruses typically circulate in wild bird populations, commonly infecting waterfowl and also regularly entering commercial poultry flocks. Live poultry markets provide an ideal environment for the spread AI and potentially the selection of mutants with a greater propensity for infecting humans because of the potential for spill over from birds to humans. Pathology from these AI virus infections is associated with a dysregulated immune response, which is characterized by systemic spread of the virus, lymphopenia, and hypercytokinemia. It has been well documented that host/pathogen interactions, particularly molecules of the immune system, play a significant role in both disease susceptibility as well as disease outcome. Here, we review the immune/virus interactions in both avian and mammalian species, and provide an overview or our understanding of how immune dysregulation is driven. Understanding these susceptibility factors is critical for the development of new vaccines and therapeutics to combat the next pandemic influenza.

## Emergence of Avian Influenza (AI) Virus Infection in Humans

Influenza A viruses have consistently posed a major threat to human health, both through seasonal infections and pandemic outbreaks (1). AI viruses have contributed significantly to this, and in recent years, highly pathogenic AI (HPAI) viruses have emerged as a major zoonotic threat. AI viruses naturally circulate in wild bird populations, including but not limited to, ducks and waterfowl, and can spill over to poultry birds such as chickens. Other than a few novel strains isolated in bats, all influenza A subtypes have been found in aquatic birds, which act as natural reservoirs for the viruses (2). These viruses typically replicate in the gastrointestinal and upper respiratory tract of both these natural hosts and chickens, and typically present as subclinical to mild disease (3, 4). Based on these clinical signs in chickens, these influenza viruses are classified as “low pathogenicity AI” (LPAI) infections (5). Although many AI viruses circulate without causing serious disease, other viral subtypes can lead to more severe outbreaks within birds. Birds infected with these subtypes have more severe pathogenesis and rapid disease progression, often as the result of dissemination of the virus into tissues peripheral to the gastrointestinal and respiratory tracts. This type of infection in chickens defines the subtypes as HPAI (5). Highlighting the importance of HPAI viruses, recent outbreaks of HPAI H5N6 in China and the Philippines have caused approximately 37,000 bird deaths and 400,000 more culled at an economic cost of nearly $USD40 million (6, 7), and novel strains of H7 viruses such as H7N4 continue to cause sporadic outbreaks (8). While such outbreaks represent a significant economic burden to the poultry industry, of greater concern is the potential for HPAI viruses to cross the species barrier into mammals, especially humans.

Despite there being a broad range of AI subtypes, fortunately, only a very select subset of these have been shown to infect humans with highly pathogenic consequences (9). The first known HPAI infections in humans were highlighted by the outbreak of H5N1 avian-derived influenza in Hong Kong in 1997, leading to 6 deaths from 18 confirmed cases (10). Since then, sporadic outbreaks of H5N1 have had highly pathogenic consequences in humans, resulting in over 450 deaths from approximately 900 cases (11–13). The emergence of the avian-derived H7N9 strain infecting humans was first described in March 2013 in China’s Yangtze River Delta, which has since caused 613 deaths out of 1,566 human cases throughout most of China as of January 2018 (14). This viral subtype is of a particular concern, as unlike H5N1, which is highly pathogenic in chickens and humans, H7N9 typically presents as an LPAI in chickens, but causes a high mortality rate in humans (40%), similar to that seen for H5N1 infections. H7N9 is one of several LPAI viruses in the H7 family capable of human infections, with viral transmission usually only acquired through close contact with host species (15–17). However, for reasons that are still unclear, H7N9 has greater transmissibility and more severe disease outcomes in humans than any other H7 viruses (18, 19). Thus, differences in clinical presentation across species, coupled with the potential of viruses such as H7N9 to cause a pandemic outbreak *via* evidence of human-to-human transmission (20), makes understanding the mechanisms by which these viruses cross the species barrier and become highly pathogenic in humans a critical area of investigation. Here, we discuss recent findings relating viral fitness to host susceptibility factors to understand how HPAI phenotypes are developed. We also outline how clinical manifestations following infection with LPAI or HPAI strains across different species provide further insights into the mechanisms underlying disease severity and susceptibility.

## Clinical Manifestations of Disease in Different Species

Human cases of AI infection have become increasingly common since outbreaks of H5N1 in the late 1990s and accentuated by a dramatic increase in H7N9 infections during the recent “fifth wave” of epidemic infections in China (21–23). These viruses were commonly contracted by people in regular close contact with live poultry markets (24, 25), where outbreaks of AI viruses in chickens can be common yet go largely unnoticed, especially in the case of H7N9 infections. Despite the vast diversity of AI viruses, predominantly only viruses from three hemagglutinin (HA) subtypes have been recorded to naturally infect humans: H5Nx viruses, most notably H5N1; H7Nx viruses such as H7N9; and H9Nx viruses, commonly H9N2. H9Nx strains present as LPAI infections in birds and have less severe symptoms in human hosts compared to H5 and H7 strains (26–29). Despite this, as H9N2 viruses are regularly found co-circulating with H5N1 and H7N9 with assortment frequently occurring between these viruses in poultry there is a real possibility of a novel HPAI strain emerging from these subtypes (30, 31). While there have been isolated cases of human infections with other subtypes, such as H10 (32), and H6 viruses which have been discussed as a potential precursor to H5N1 with pandemic potential (33–38), H5/H7/H9-subtypes of AI remain of greatest concern for human infections and potential pandemics. Thus, understanding the clinical manifestations of these viruses in avian hosts is needed for our understanding of why these viruses are considered such a threat.

In the case of LPAI, infections tend to localize in the mucosal surfaces of the gastrointestinal tract of infected birds and although often asymptomatic, chickens may present with mild clinical signs following infection. These include excess mucus and congested tracheae, watery droppings, and mild respiratory inflammation, with rarely any other signs of respiratory disease associated with influenza infections (3, 26, 39, 40). Highest viral titers typically occur 2–3 days after infection with limited gross lesions evident, allowing the virus to replicate and be excreted into the environment with little to no effect on the host animal (41, 42). Interestingly, ducks can exhibit even more limited clinical signs following LPAI infection than chickens (43). Thus, it is interesting to note that even in avian hosts, there is a range of clinical severity to LPAI viruses, with chickens showing more moderate/severe signs compared to waterfowl infected with LPAI.

In contrast to LPAI viruses, HPAI viruses have the ability to induce severe disease and cause devastating outbreaks with high mortality rates in poultry (44, 45). In chickens, H5N1 causes acute illness with high levels of viral shedding and clinical signs such as dehydration, nasal discharge, and lesions in many tissue types (46–48). While LPAI infections tend to remain within the fecal–oral tract of infected birds, HPAI infections are often identified by virus spreading systemically to multiple tissues (49). Suzuki and colleagues (50) described the pathology of H5N1 infections in chickens, finding that clinical signs progressed from milder signs such as feather ruffling and depression behavior, to the more severe outcomes of hemorrhaging and edema in multiple tissues. These birds also showed severe respiratory distress not seen in LPAI infections (50). By contrast, ducks can present with a wider range of symptoms following infection with HPAI H5N1, with experimentally infected ducks having clinical signs as mild as depressive behavior without any other complications (51). Ducks may also show severe signs such as those seen in chickens, with common outcomes including neurological spread and hemorrhaging in the body extremities. In addition, Yamamoto and colleagues have also found that domestic ducks, unlike chickens, show corneal opacity following H5N1 infection and less severe hemorrhaging compared to chickens (52, 53). Likewise, wild ducks have been shown to exhibit less severe signs following H5N1 infection compared to other gallinaceous birds including domestic ducks, despite showing high levels of viral shedding consistent with an HPAI infection, which may suggest their role as a key reservoir species (48).

Avian influenza viruses also have the ability to infect pigs, which are housed in close proximity to human populations. Pigs often act as a “mixing vessel” for influenza viruses, which are able to reassort and thus infect humans (54, 55). While this is particularly the case for low pathogenicity viruses, there has been little evidence to suggest that pigs can contract highly pathogenic strains such as H5N1 and H7N9 to any great level, with H5N1 strains isolated from pigs in China found to be attenuated from the HPAI form (56). Although there have been no confirmed cases of H7N9 infection in pigs (57), H7N9 can replicate, cause pathology, and transmit among pigs during *in vivo* studies at low levels (58–60), as well as replicate in swine respiratory tissue *in vitro*, reinforcing the idea that pigs could still act as an important reservoir species for mammalian-adapted H7N9 (61). Of particular concern, reports of H7N2 infection in pigs (62) suggest that as the frequency of H7Nx cases increases, the likelihood of an H7N9 virus infecting pigs and potentially gaining stable mammalian transmissibility is a genuine possibility.

Studies in ferrets as a model for human infection have shown that mammalian-adapted AI viruses typically localize to the respiratory tract (63), however, these viruses have can have a limited ability to transmit *via* droplets (64–66). H5N1 viruses cause acute illness in the upper respiratory tract of ferrets with symptoms such as nasal discharge, high temperatures, and weight loss due to dehydration, and worsened pathogenesis, as highlighted by lung damage due to extensive infiltration of the lung tissue by inflammatory cells (18, 67, 68). H7N9 can also cause severe respiratory distress in this way, with lengthened time until viral clearance contributing to viral transmission and the substantial inflammation in the lungs of ferrets (69). Severe infections may cause complications such as viral pneumonia due to the breakdown of lung endothelial barriers, which contributes to this systemic spread and may lead to encephalitis or other neurological issues (18, 70). However, systemic spread to the central nervous system is more commonly associated with HPAI H5N1 than LPAI H7N9 (68).

**Figure 1:**
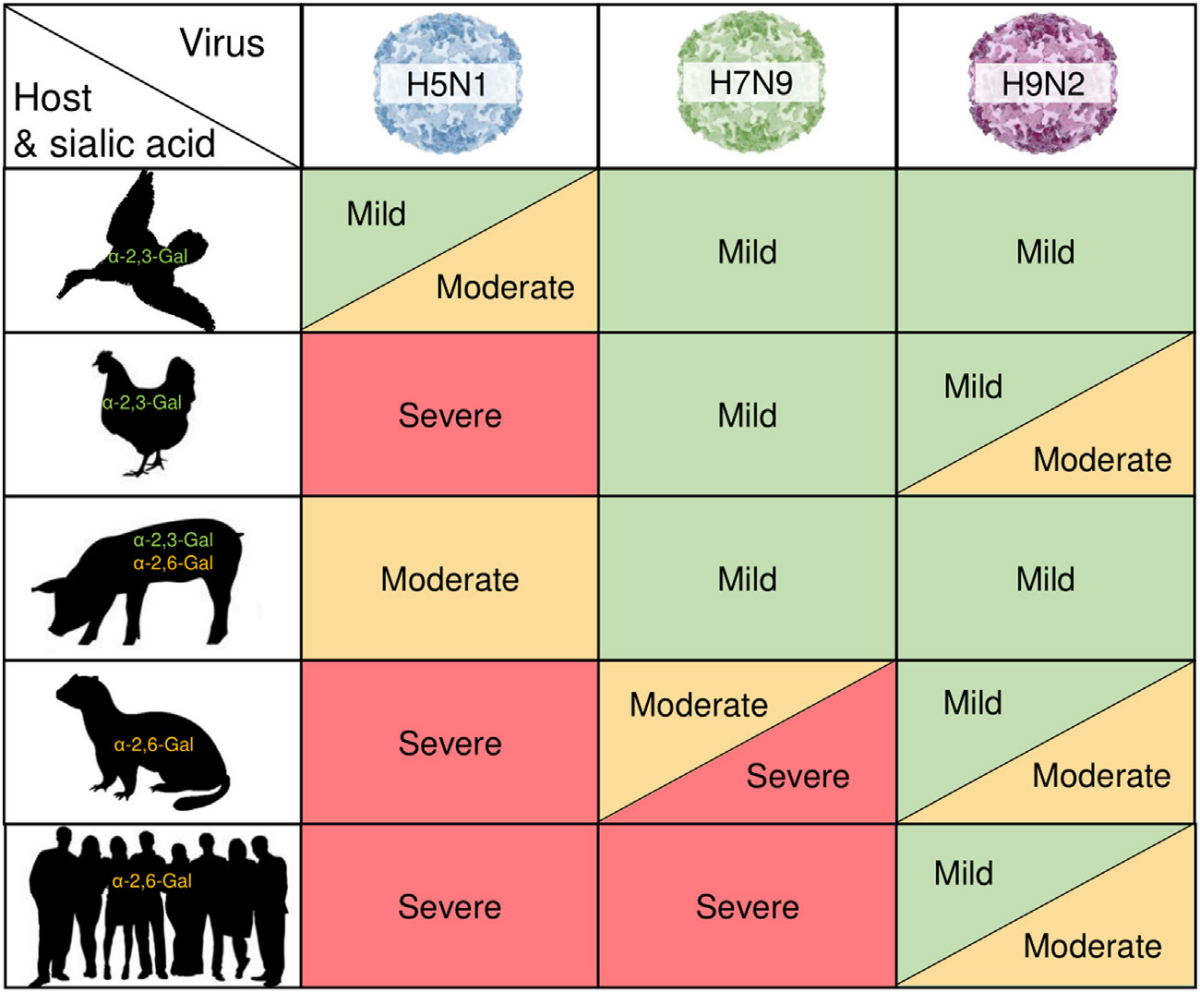
Zoonotic influenza pathology is linked to host biology. H5N1, H7N9, and H9N2 avian influenza (AI) viruses causing varying degrees of clinical manifestations across different species is shown against different host and their sialic acid properties. AI viral hemagglutinin proteins selectively and preferentially bind to sialic residues with either an α-2,3-Gal or α-2,6-Gal terminating sequence on the host cell surface to allow for viral fusion and entry. This represents viral fitness and is a potential mechanism underlying the degree of disease severity and susceptibility in the different hosts.

Symptoms following human infection with AI are similar to those observed in the ferret model. Less severe cases present as more typical influenza infections, with symptoms such as fever and coughing among those commonly associated with influenza-related illness (16, 71). However, these viruses can cause severe respiratory illness following infection in the lungs, which may manifest as atypical viral pneumonia and acute respiratory distress syndrome, and often patients who have contracted these infections die from respiratory failure (72, 73). Furthermore, much like in birds, dissemination of the virus away from the site of infection leads to other complications such as organ failure, encephalitis, and internal bleeding due to tissue destruction (10, 71, 74), all of which contribute to the lethal nature of these viruses. A summary of the varying degrees of clinical manifestations between the different species is depicted in Figure 1. These trends, including the ability for an LPAI AI viruses such as H7N9 to cause severe fatal disease in humans, is therefore of great importance to understand the pathology driving the variable signs and symptoms of these infections across species to best combat future infections.

## Pathogenesis of HPAI Infections

Several factors appear to contribute to the worsened pathology observed in HPAI infections when compared with LPAI infections. One hallmark of HPAI pathogenesis is a rapid and robust cytokine response, often referred to as a “cytokine storm” or hypercytokinemia. This build-up of cytokines causes an inflammatory environment at the site of infection, leading to immune cell infiltration. In addition to the hypercytokinemia, there is a well-established loss of leukocytes, leading to the severe pathogenesis seen in these infections (75). Recently, the main factors associated with pathology were further described by Kuchipudi and colleagues (76). Following infection with H5N1 *in vitro*, chicken lung cells had increased expression of specific pro-inflammatory cytokines, particularly interleukins (IL)-6 and IL-8 when compared to cells infected with LPAI H2N3, suggesting that IL-6 and IL-8 may be key regulators leading to worsened pathology of HPAI compared to LPAI (76). IL-6 and IL-8 were also found to be significantly upregulated in the lungs of H5N1 infected ferrets, as well as in peripheral tissues, including the spleen, heart, and liver (77). Interestingly, this study also found that IL-6 and IL-8 were downregulated in the nasal turbinates following infection with pandemic H1N1 virus, which produced a less severe clinical infection compared to the H5N1 in ferrets (77). These findings were consistent with studies completed in rhesus macaques, which similarly showed upregulation of tumor necrosis factor α (TNFα), IL-6, and IL-8 in response to experimentally induced H5N1 infection, along with an increase in the antiviral interferons (IFNs), findings which correlate to the severe fever symptoms observed in the macaques at the peak of the fever response at day six post-infection (78). Moreover, infections with the recently emerged HPAI H5N6 in chickens, which caused human fatalities, was shown to have a very distinct immune response compared to other H5N6 strains by producing much higher levels of IL-6, IL-8, and other pro-inflammatory mediators such as TNFα compared to previously identified strains (79). While chickens and ferrets show similar pathogenesis, conversely, duck lung cells infected with the same viruses showed a decrease in IL-6 expression compared to the LPAI viruses, while IL-8 remained unchanged. It suggests that IL-6 may play a pivotal role in the regulation of AI pathology in birds and, as previously discussed, ducks typically show lessened disease severity following H5N1 infection compared to chickens (Figure 1). Moreover in humans, H5N1 elicits a similarly robust cytokine response, with the upregulation of IL-6, IL-10, and TNFα in response to H5N1 (80).

As the H7N9 virus is classified as an LPAI in chickens, it is interesting to note that a similar trend was observed when H7N9 of human origin were shown to induce increased production of pro-inflammatory IL-6 and IL-8 cytokines when compared to H7N9 of chicken origin (81). In the same study, it was demonstrated that IFNλ1 production was reduced for the human isolate, suggesting a possible modulation of the immune response. In addition, Wu and colleagues also found that H7N9 patients had higher levels of C-reactive protein expression in their plasma compared to H1N1 patients, and as C-reactive protein is associated with broader inflammatory responses, it suggests that this may be yet another factor alongside these regulatory cytokines contributing to disease severity (82). A similar pro-inflammatory response was observed when alveolar macrophages were infected with H7N9, however, when compared to H5N1, this cytokine response was demonstrated to be milder (83). Downregulation of these inflammatory responses to infection confers a level of immunity to these viruses in pigs, which hints at how pigs can act as mixing vessel species for AI without succumbing to severe disease. Human lung epithelial cells can express 100-fold higher levels of TNFα compared to pig lung epithelia, with suppressor of cytokine signaling 3 (SOCS3) identified as a key factor in reducing levels of TNFα in pig cells (84).

The drivers of cytokine production are resident and infiltrating immune cells, which release these cytokines in response to the infection to recruit other immune cells and hinder viral replication. This occurs following cellular activation and results in further activation of leukocytes recruited to the area in a positive feedback loop (85). As pro-inflammatory molecules are associated with apoptotic pathways in humans, increased cytokine production is likely to be a contributing factor in the loss of immune cells, or leukopenia, observed in severe cases of HPAI infection (55). These pro-inflammatory regulators lead to upregulation of the death signaling molecule Fas-ligand on the infected host cell to initiate the caspase-mediated Fas-associated pathway, in which Fas receptors on the immune cell (part of the TNF-receptor family) bind to Fas-ligand and subsequently recruit the Fas-associated death domain (FADD) molecule (86). FADD interacts with caspase-8, which initiates a signaling cascade within the immune cell, resulting in the destruction of cellular components and thus cell death, which may be causative of the pathology seen in influenza patients (87). Indeed, both H5N1 and H7N9 have been observed to cause leukopenia in hospitalized patients (88–90). According to Boonnak and colleagues, CD8^+^ T cells can be particularly affected by the Fas-ligand mediated pathway, where Fas-ligand was upregulated on plasmocytoid dendritic cells during lethal H5N1 infection in mice, which then lead to apoptosis of influenza-specific CD8^+^ T cells in the lung draining lymph nodes (86). Furthermore, in hospitalized H7N9-infected patients during the emergence of H7N9 in 2013, the persistence of immune cell subsets within the blood contributed to disease severity and fatal outcomes, with the continuation of CD38^+^HLA-DR^+^ CD8^+^ T cell responses shown to be predictive of fatal outcomes, possibly due to longer-lasting inflammatory responses in the peripheral blood and lung (91). Loss of peripheral blood lymphocytes was also observed in human seasonal influenza A infections, with these cell subsets succumbing to apoptosis by the same apoptotic pathways described for AI viruses (92). In summary, LPAI versus HPAI viral strains differentially promote the induction of pro-inflammatory cytokines, which then alter disease outcomes in different species. The potential mechanisms by which certain HPAI AI viruses cause more severe disease will be further discussed in the following sections.

## Viral Fitness Across Species

In order for AI viruses to be capable of infecting multiple host species they require viral adaptations allowing replication in different host cells, which ultimately increase their genetic fitness and create a sustainably replicating and transmitting virus (93). It is well established that for zoonotic transmission of AI viruses, the virus needs to present the appropriate HA binding specificity to allow viral fusion and entry. AI virus HA proteins typically have a binding preference for sialic acid residues with an α-2,3-Gal terminating sequence found on the surface of avian cells (Figure 1), resulting in restriction to avian cells (63, 94). Therefore, for AI viruses to gain entry into human cells displaying an α-2,6-Gal terminating sequence, modifications to the HA binding site may be required to allow this new interaction.

One of the commonly associated amino acid substitutions for LPAI strains converting from avian to mammalian receptor-specificity is a HA Q226L substitution (58, 95–97). For example, LPAI H9N2 isolates from birds can adopt a Q226L substitution, which increases its mammalian receptor binding affinity and potentially infect mammalian hosts (98, 99). However, with regards to H7N9 human isolates, Belser and colleagues described the Q226 bearing Shanghai/1 and the L226 bearing Anhui/1 as binding to largely avian α-2,3-Gal receptor analogs and mixed α-2,3/α-2,6-Gal receptors, respectively (64). Despite these differences, infections with each isolate produced effective replication in the lower respiratory tract of ferrets, suggesting additional factors are involved in mammalian adaptation. Similarly, this Q226L mutation has not been commonly observed in the HA of H5N1 HPAI viruses (100, 101). However, alternative substitutions, HA Q192H and HA I151T, can confer increased ability for replication in human hosts. Moreover, Herfst and colleagues demonstrated a closely related change to the HA of H5N1, HA Q222L, conferring more efficient replication and transmission in the ferret model (66). Additional to this HA mutation, an E627K change in the polymerase basic 2 (PB2) protein in H5N1 HPAI was also shown to be critical for transmission in ferrets (64, 102–105).

In addition to HA-sialic acid binding, key to viral fitness is the presence of a multi-basic cleavage site (MBCS), which has been shown extensively with H5N1, that the presence of an MBCS often dictates the use of the term HPAI (106, 107). The presence of an MBCS in the HA of influenza A viruses allows HA cleavage by additional enzymes such as furin-like proteases, whereas without an MBCS, the virus relies on only trypsin-like proteases (108). This flexible range of enzyme activity results in the virus being able to infect a greater range of cells and can lead to systemic infection (106, 107, 109). Though commonly associated with H5N1, this motif is seen in other avian-infecting HPAI viruses such as H7N3, however, these strains are less frequently transmitted to humans (19, 110). Interestingly, LPAI can also acquire MBCS motifs, changing the pathogenicity of the virus from low to high, such as in the case of an H7N8 outbreak in Turkey in 2016, where an LPAI virus caused a severe outbreak in the poultry due to the spontaneous addition of an MBCS, leading to over 800 bird deaths (111). However, the addition of an MBCS does not guarantee an LPAI virus to increase its pathogenicity, as recombinant H5 and H7 viruses do not exhibit HPAI pathology in chickens specifically due to the addition of an MBCS (112), which suggests that these motifs are one of many factors contributing to HPAI pathogenesis. H7N9 has also been shown to be able to obtain an MBCS to become highly pathogenic in chickens (113). Imai and colleagues showed increased disease severity of an MBCS-containing H7N9 virus in the ferret model compared to an LPAI H7N9 virus (18). However, H7N9 will still cause severe disease without an MBCS in most human cases, highlighting how unique this virus is in the AI landscape for its ability to show HPAI-like symptoms in mammals, while maintaining low pathogenicity in birds. Similarly for H5Nx viruses, several additional changes in the H5 HA protein, such as an N158D mutation, also allow greater replication of the virus in ferrets without the need for an MBCS, which combined with reassortment with human-adapted H1N1 gene segments shows the potential for these viruses to not only cross into humans but also cause severe, sustained human infection without systemic spread (114). This, coupled with H7N9’s ability to cause severe disease without the requirement of an MBCS, suggests that while the MBCS still acts as a key virulence factor for AI viruses, it is not a sole-determining factor for HPAI in humans.

A key interaction between host and viral proteins is the interplay between Mx GTPases and viral nucleoprotein (NP), which may be pivotal in determining viral fitness. Human MxA and murine Mx1 protein have been shown to confer antiviral protection against influenza A viruses by interfering with the ability of NP to localize to the nucleus, inhibiting the viral replication cycle (115). While many influenza viruses are susceptible to Mx restriction, changes in the NP have been shown to confer resistance to this form of protection, particularly in the case of AI viruses such as H5N1 which shows greater susceptibility to MxA inhibition than pandemic H1N1 (116, 117). Moreover, LPAI H7N9 viruses were similarly shown to be affected by Mx1 in infected mice compared to H5N1 by Deeg and colleagues, who showed that these viruses required human adaptive motifs in their NPs to evade Mx restriction (118). Interestingly, avian species such as chickens have been found to have an Mx that does not display strong antiviral properties, suggestive of why these avian viruses do not commonly have NP capable of MxA evasion (119, 120). Therefore, while these AI viruses often appear to cause worsened disease progression due to their differences to human-infecting strains, in the case of Mx restriction it is a lack of human adaptation that may provide a level of protection to mammalian hosts.

Another key virulence factor elucidated in recent years is the ability of the non-structural protein 1 (NS1) to aid viral escape in the host immune system. In avian hosts, NS1 has been associated with worsened pathology through increases in iNOS and oxygen-reactive species for both LPAI H9N2 (121) and HPAI H5N1 (122). However, in contrast in mammals, the NS1 protein has been more closely associated with inhibition of host IFN responses. Jia and colleagues (123) showed that the H5N1 NS1 protein inhibits IFN production through interference with the JAK/STAT pathway. They found that expression of NS1 in HeLa cells prevented STAT phosphorylation and upregulated inhibitors of this pathway to prevent expression of IFNAR and SOCS3 proteins, which generally upregulate IFN expression (123). Furthermore, a naturally occurring deletion in the H5N1 NS1 effector domain can attenuate the virulence of the virus in both chickens and mice, suggesting that this protein is critical in the ability of H5N1 to suppress host immune antiviral responses across hosts (124). The pathogenicity of H5N1 in mice is also affected by the NS1 protein, as a single mutation (P42S) conferred greater pathogenicity to the virus by preventing nuclear factor-κB (NF-κB) and interferon-regulatory factor 3 (IRF3) signaling, and thus inhibiting IFN responses (125). Interestingly, in cats the NS1 protein can be associated with blocking NF-κB and IRF3 signaling in response to the emerging HPAI H5N6 virus, with inhibition of the IFN-β promoter blunting the feline IFN response (126), suggesting that NS1 may have different ways of interacting with influenza hosts across species to produce similar immune suppression. On the other hand, Thube and colleagues investigated the IFN responses of HPAI H5N1 compared to LPAI H11N1 and suggested that decreased IFN signaling occurred independently of NS1, suggesting other viral elements can also induce a reduced antiviral state in cells (127). It is worth noting that while the NS1 of LPAI H7N9 is inefficient at binding to CPSF30 (involved in pre-mRNA processing), a single I106M mutation restores CPSF30 binding to NS1 thereby blocking the expression of host antiviral genes. This renders the virus more virulent than other LPAI infections (128), which may explain why H7N9 causes more severe disease in humans compared to other LPAIs. These results also highlight inhibition of IFN-activation pathways as an important viral factor in preventing host immune responses to infection, allowing for more productive infection and potentially more severe clinical outcomes.

## Host Susceptibility Factors

In addition to the ability of the virus to gain function through mutation, in recent years there has been an increasing focus on how host genetic factors can lead to changes in resistance or susceptibility to influenza A viruses. While many factors have been identified in preventing influenza A infection, a key which has come to light for AI host/pathogen interactions is the interferon-induced transmembrane (IFITM) protein family, which unlike other factors such as MxA seems to be predominantly the host, rather than the virus, that seems to control whether the virus is able to replicate. IFITMs are family of transmembrane antiviral proteins that are stimulated by the presence of elevated IFN levels, giving another reason why so many AI viruses attempt to quash the IFN response (129, 130). The IFITM proteins can interfere with viral entry to the cytosol *via* cell membranes (131), with Brass and colleagues showing that overexpression of human IFITMs 1, 2, and 3 effectively blocked infection with several influenza A pseudoviruses (retroviruses expressing influenza surface proteins), including those enveloped with H5 and H7 proteins (132). Moreover, IFITM3 specifically localizes to the endosomes due to phosphorylation of the Y20 tyrosine residue, enabling these proteins to intrinsically target pH-dependent viral pathways such as that seen with influenza A viruses (133).

The IFITM3 molecule can play a significant role in mice and human influenza infections. Mice inoculated with influenza antigen showing higher IFITM3 expression in the lungs developed a more robust lung tissue-resident memory CD8^+^ T cell response as well as a longer duration of response even following reduction of IFN-α, suggestive of this molecule playing a role in not only innate immunity but also adaptive immunity as well (134). Of particular note, a single-nucleotide polymorphism (SNP) in the IFITM3 gene, *rs12252-C*, has been shown to strongly correlate to worsened disease progression, as this SNP leads to a truncated splice-variant that affects the protein’s ability to localize to the membrane (135, 136). IFITM3 protein dysfunction can be associated with severe hypercytokinemia and worsened disease progression in H7N9-infected hospitalized patients. In support, Zhang and colleagues showed that the *rs12252-C* mutation correlated to severe seasonal H1N1 influenza cases in Chinese populations where the mutation appeared in higher frequencies (136, 137). However, studies have shown that while this SNP does affect IFITM3′s ability to localize, restriction of the virus may continue with the variant protein or with a Y20A mutation to affect the localization of the full protein (138). Furthermore, a recent study by Makvandi-Nejad and colleagues found that primary cell lines homozygous for the *rs12252-C* SNP expressed the non-truncated mRNA transcript and thus expressed the wild-type IFITM3 protein at levels greater than 99% when compared to the truncated versions (139), which may provide insights into why the *rs12252-C* mutation appears not to act as a significant risk factor in Caucasian populations, but perhaps in the Chinese patients. Moreover, an additional SNP has recently been identified, *rs34481144-A*, which affects the promoter for the *IFITM3* gene, resulting in lower IFITM3 expression compared to hosts without the mutation. Interestingly, both the *rs12252-C* and *rs34481144-A* mutation were found to be non-overlapping, as the risk allele for one was inherited with the protective allele of the other, suggesting a multifaceted IFITM response to influenza A viruses (140).

IFITM1 and IFITM3 distribution in mice has been investigated in the context of H9N2 infection, with the distribution of these proteins correlating with increased restriction of the virus’ entry into host tissues, due to upregulation in the lungs and peripheral tissues of BALB/c mice following inoculation. Interestingly, when infected with a H9N2 strain with higher pathogenicity and ability for systemic spread due to a K627E mutation (rV_K627E_) in the viral PB2 protein, compared to the wild-type strain, IFITM3 was upregulated accordingly in the brain of rV_K627E_-infected mice to combat this virus’ ability to cause viral encephalitis (141). IFITM3 responses to H5 and H7 proteins have also been assessed in pigs and bats, with both these HA subtypes showing restricted entry due to the action of these IFITM3 proteins (142), suggestive of the broad action of IFITM molecules across species known to contract and potentially disseminate AI viruses. That IFITMs restrict AI viruses in “mixing vessel” species (i.e., pigs and bats) suggests that these proteins may have a key role in preventing the spread of human-infecting AI viruses through these routes, as well as contributing to these species showing lessened pathology compared to other mammalian species.

The role of IFITMs against AI viruses in avian species has not been clearly defined, though a study by Smith and colleagues has shown that chicken IFITM3 (which is “human IFITM1-like”) similarly restricts H5 and H7 expressing viruses (143). However, when chickens were infected with H5N1, expression of IFITM molecules was not highly upregulated compared to other human-infecting seasonal strains such as H1N1, with IFITMs showing the weakest inhibiting effect against H7N9 (144). Hence, IFITM expression in chickens appears to be limited and does not vary greatly whether the virus is of low or high pathogenicity. Conversely, the IFITM expression profile observed in ducks is far more robust and variable between viral strains, whereby infection with LPAI H5N2 virus was reported to consistently cause a 3-fold increase in IFITM1, 2, and 3 expression levels in the lungs and ileum on day one post-inoculation, while infection with the HPAI H5N1 virus caused up to a 93-fold increase in IFITM3 expression in the lungs in a similar time frame (145). Therefore, understanding the differences in variable IFITM expression, and the reasons why chickens mount a lesser IFITM response to influenza viruses, may prove pivotal in understanding why some birds succumb to HPAI infection while others survive.

**Figure 2:**
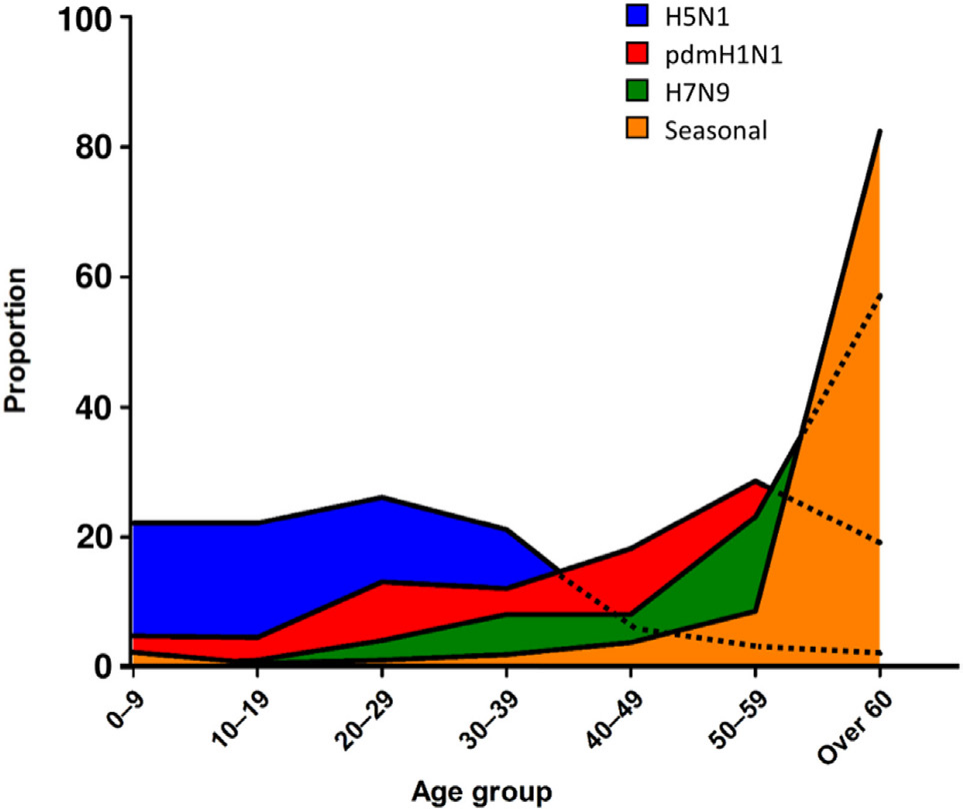
Age-related mortality trends highlight impact of host–pathogen relationships. Frequency of age groups of patients who succumb to different strains of influenza is graphed as a proportion of total fatalities for a given strain. When we assessed the age of patients who succumb to different strains of influenza, as a proportion of the total mortalities for a given strain, trends emerge as to the host susceptibilities. For seasonal influenza, older patients (>60 years old) were the most susceptible, however, for a variation on seasonal influenza, pdmH1N1 2009, the age of patients who succumbed was reduced and included significant mortalities between 20 and 59 years old. Interestingly, the highly pathogenic AI H5N1 was predominantly fatal in those under 40 years old, whereas H7N9, a low pathogenicity AI strain, followed a similar trend to seasonal influenza.

In addition to specific IFITM mutations, broad immunodeficiency can lead to worsened pathology and disease outcomes in humans and animals. For example, immunocompromised patients who contract LPAI viruses such as H9N2 suffer severe respiratory distress, and though many of these LPAI remain mild even in immunocompromised patients, H9N2 induces stronger cytokine responses than seasonal influenza viruses (146, 147). These trends are also observed in avian hosts, in which Nili and Asasi found that chickens co-infected with other pathogens such as *M. gallisepticum* showed worsened clinical signs such as severe necrotizing tracheitis, leading to a 19% mortality in the flock (148, 149). Therefore, it is apparent that both the host and pathogen can contribute to perturbed inflammation and severe disease outcomes. In addition, in humans, more severe AI subtypes have been associated with mortalities in very distinct age demographics when compared to seasonal influenza (Figure 2), and often manifest in age groups not commonly associated with immunodeficiency. Fatal cases in children (0–9 years) and younger adults (10–19 years) were predominantly caused by HPAI H5N1 infection, with 80.3% of H5N1 fatal cases seen in people aged 35 or under (12). Interestingly, a similar trend was observed with the pandemic [pdmH1N1(2009)] strain, which led to increased infection in younger age groups compared to other seasonal strains circulating at the same time (150). An interesting observation from the 2009 pandemic, however, was that disease outcomes were more mild in newly weaned ferrets infected with pdmH1N1(2009) as well as in younger children, suggesting the immune response in younger individuals may have a protective response to this strain (151, 152). Conversely, seasonal strains of influenza disproportionately impact older ferrets infected, which display a greater degree of morbidity and reduced HA and T cell responses (153). This is also true of human patients also whereby older patients (>60 years of age) are most susceptible to seasonal influenza strains (80% of mortality cases). With regards to H7N9 LPAI infections, the highest mortality was also skewed toward older people, which is likely due to the propensity for H7N9 cases to be found in live poultry farmers, typically older men (154, 155). This emphasizes the dynamic relationship between the pathogen and the human host, and how different strains of influenza viruses can lead to differential fatal outcomes across different ages, a concept further explored by Gostic and colleagues who suggest that pre-immunity to these viruses may confer differing levels of protection to AI based on whether they have been exposed to the viral HA class while still young (156).

While IFITM proteins aim to restrict viral entry, the interaction between influenza virus peptides and major histocompatibility complex (MHC) molecules is a key host–pathogen interaction affecting the outcome of disease following initial infection. In humans, MHC molecules are encoded for by the human leukocyte antigen (HLA) system, with a vast array of alleles in the genes encoding for the molecules responsible for the recognition of antigenic peptides. As such, different HLA subtypes confer different levels of susceptibility to influenza A viruses, as not all HLA subtypes respond to influenza peptides in the same way. For example, human populations expressing HLA-A*02:01 can elicit strong, cross-protective CD8^+^ T cell responses following presentation of the internally conserved M1_58–66_ epitope (157–160); this epitope is one of the most immunogenic influenza peptides observed in humans with HLA-A*02:01 being the most common HLA alleles expressed worldwide (157). Moreover, individuals lacking common HLA alleles may be at greater risk of influenza A infections, such as those carrying the HLA-A*24:02 allele, associated with increased mortality in individuals infected with pandemic H1N1 virus (161). Indigenous populations in particular are susceptible to influenza A due to a lowered prevalence of protective HLA variants toward these viruses (160). Wang and colleagues suggest that for AI viruses such as H7N9, MHC-interactions with internally conserved epitopes from other influenza A strains, such as pandemic H1N1, may explain why some populations show greater immunity through cross-reactive CD8^+^ T cell responses than others (72). However, some H7N9 peptides may have cross-conservation with human host proteins that the immune system recognizes as “self,” leading to an attenuated immune response to the virus and thus worsened disease progression due to T cell-mediated tolerance (162).

**Figure 3:**
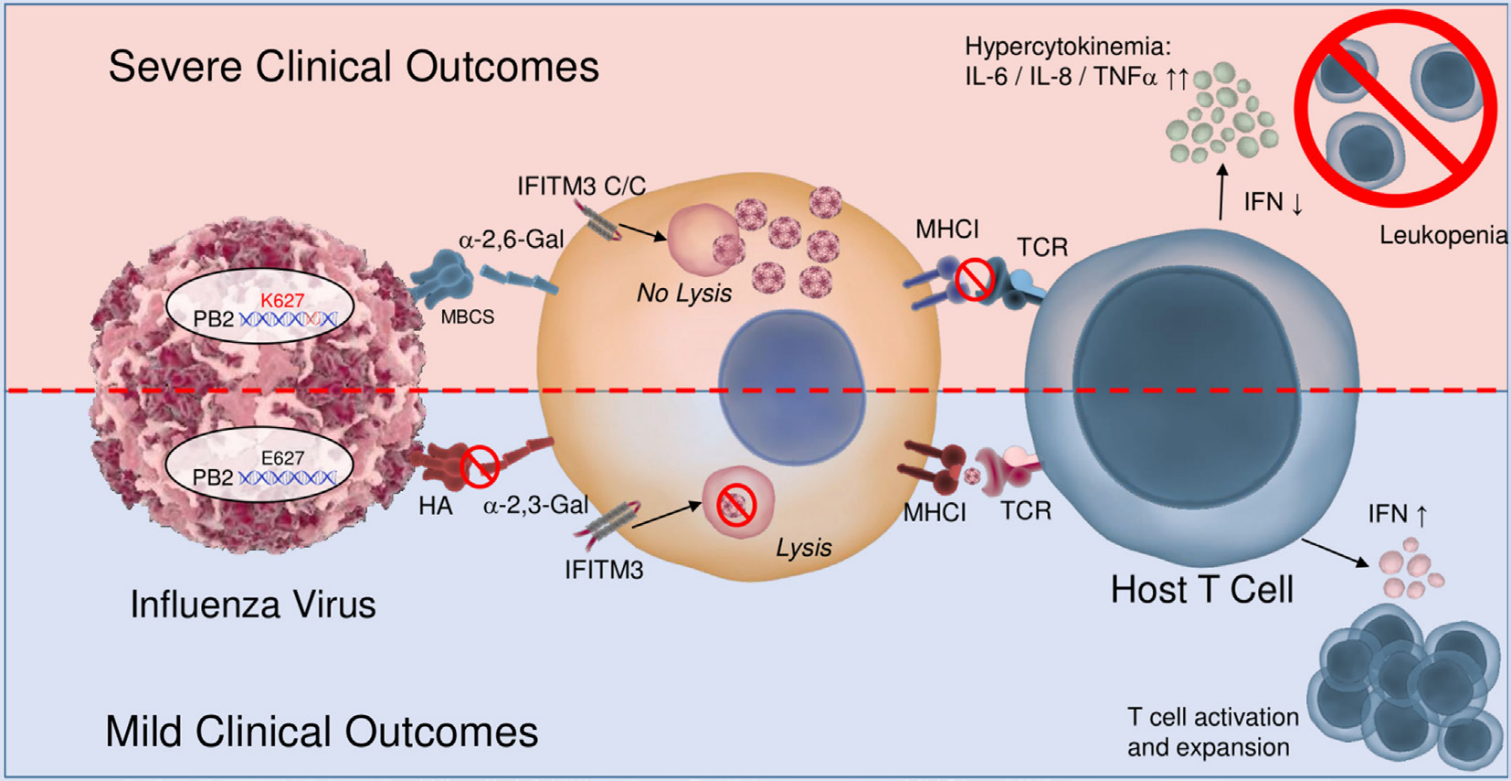
Mechanisms leading to severe clinical outcomes involve both host and pathogen elements. A number of factors have been elucidated to contribute to the severity of influenza disease outcomes. This include viral fitness elements, such as the presence of a multi-basic cleavage site (MBCS), mutations to the polymerase basic 2 (PB2) genes, as well as the presentation of hemagglutinin (HA) protein capable of binding to human sialic acids. These viral fitness elements work in concert with host factors such as modified interferon-induced transmembrane (IFITM) proteins, with reduced ability to combat the virus and MHCI diversity which can or cannot present the antigen appropriately and efficiently to host T cells. Ultimately, these and other elements lead to changes in production of key cytokines as well as cellular activation that drives inflammation, cell death, and clinical manifestation of disease.

Interestingly, chickens show a restricted repertoire of MHC alleles compared to other species, and these haplotypes themselves are poorly characterized. As such, a few studies have been conducted into characterizing T cell epitopes in response to H5N1 infections, one such study predicting 25 potential T cell epitopes in the NP of H5N1 in four haplotypes (163). More recently, experiments into epitopes of a specific haplotype, BF2*15, further characterized NP epitopes that may lead to protective immunity against H5N1 (164). This limited repertoire may explain why chickens show more severe clinical outcomes due to HPAI such as H5N1 compared to waterfowl, as ducks show extensive diversity in their MHC class I alleles which allows the immune system greater coverage for viral variation (165). A recent investigation into duck MHC class I molecules found that the duck *Anpl*-UAA*01 complex showed similar peptide binding properties to HLA-A*02:01 in humans and as such appears to cover a greater array of influenza A virus epitopes compared to similar chicken MHC molecules such as BF2*2101 (166). Furthermore, migratory shorebirds which act as reservoirs for AI viruses (in particular LPAI H9N2) show increased diversity in their MHC alleles, likely as a mechanism for protecting against foreign pathogens that may be encountered during migration. A study in red knots found high MHC diversity with 36 alleles detected across eight birds, which when correlated to their low prevalence of shed AI virus and high antibody titers to AI viruses, they could mount effective immune responses toward these viruses, possibly *via* cytotoxic T lymphocyte responses recognizing novel peptide/MHC complexes (167). Based on a culmination of human and animal evidence, the interactions between the various pathogen and host factors contributing to human influenza severity are summarized in Figure 3.

## Conclusion

The emergence of AI viruses is of major concern to the avian and human population. The lack of pre-existing antibody immunity and their ability to cause severe disease through multiple host and viral mechanisms makes these viruses difficult to counter. Currently, these viruses are yet to effectively replicate and transmit between humans, however, experiments in ferrets show that only a few mutations are needed for H5N1 and H7N9 viruses to quickly adapt and become a major pandemic threat (114, 168). Their ability to pass from birds to mammals commonly in contact with humans requires constant surveillance across all known bird reservoirs to limit the potential threat of an AI-derived pandemic. Characterization of the interactions between AI viruses and their hosts and how they illicit different degrees of clinical manifestations across species is of utmost importance. Here, we extend our current knowledge of the “cytokine storm” model of AI pathogenesis and delve into more complex underlying viral and host genetic factors that may also contribute greatly to disease severity and susceptibility outcomes.

## Author Contributions

WH and DL wrote and edited the manuscript. AB, TN, and KK edited the manuscript.

## Conflict of Interest Statement

The authors declare that the research was conducted in the absence of any commercial or financial relationships that could be construed as a potential conflict of interest.

## References

[1] Ehrhardt C Seyer R Hrincius ER Eierhoff T Wolff T Ludwig S Interplay between influenza A virus and the innate immune signalling. Microbes Infect (2010) 12:81–7. 10.1016/j.micinf.2009.09.007 19782761

[2] Tong S Zhu X Li Y Shi M Zhang J Bourgeois M New world bats harbor diverse influenza A viruses. PLoS Pathog (2013) 9(10):e1003657. 10.1371/journal.ppat.1003657 24130481 PMC3794996

[3] Xing Z Cardona CJ Li J Dao N Tran T Andrada J Modulation of the immune response in chickens by low-pathogenicity avian influenza virus H9N2. J Gen Virol (2008) 89(5):1288–99. 10.1099/vir.0.83362-0 18420808

[4] Daoust PY Kibenge FS Fouchier RA van de Bildt MW van Riel D Kuiken T. Replication of low pathogenic avian influenza virus in naturally infected mallard ducks (*Anas platyrhynchos*) causes no morphologic lesions. J Wildl Dis (2011) 42(2):401–9. 10.7589/0090-3558-47.2.401 21441193

[5] Alexander DJ. An overview of the epidemiology of avian influenza. Vaccine (2007) 25:5637–44. 10.1016/j.vaccine.2006.10.051 17126960

[6] Du Y Chen M Yang J Jia Y Han S Holmes EC Molecular evolution and emergence of H5N6 avian influenza virus in central China. J Virol (2017) 91(12):e00143-17. 10.1128/JVI.00143-17 28404845 PMC5446651

[7] Visperas E Simeon LM First Bird Flu Outbreak: 400,000 to Be Culled. Manila: The Philippine Star (2017).

[8] World Health Organization. Influenza at the Human-Animal Interface: Summary and Assessment, 26 January to 2 March 2018. Geneva: World Health Organization (2018).

[9] Wang Z Loh L Kedzierski L Kedzierska K Avian influenza viruses, inflammation, and CD8+ T cell immunity. Front Immunol (2016) 7:60 10.3389/fimmu.2016.00060 26973644 PMC4771736

[10] Claas EC Osterhaus AD van Beek R De Jong JC Rimmelzwaan GF Senne DA Human influenza A H5N1 virus related to a highly pathogenic avian influenza virus. Lancet (1998) 351:472–7. 10.1016/S0140-6736(97)11212-0 9482438

[11] World Health Organization. Cumulative Number of Confirmed Human Cases for Avian Influenza A(H5N1) Reported to WHO, 2003–2017. Geneva: World Health Organization (2017). Available from: http://www.who.int/influenza/human_animal_interface/2017_10_30_tableH5N1.pdf?ua=1 (Accessed: March 21, 2018).

[12] Lai S Qin Y Cowling BJ Ren X Wardrop NA Gilbert M Global epidemiology of avian influenza A H5N1 virus infection in humans, 1997–2015: a systematic review of individual case data. Lancet Infect Dis (2016) 16:e108–18. 10.1016/S1473-3099(16)00153-5 27211899 PMC4933299

[13] World Health Organization. Influenza at the Human-Animal Interface: Summary and Assessment, 28 September to 30 October 2017. Geneva: World Health Organization (2017). Available from: http://www.who.int/influenza/human_animal_interface/Influenza_Summary_IRA_HA_interface_10_30_2017.pdf (Accessed: March 21, 2018).

[14] World Health Organization. Influenza at the Human-Animal Interface: Summary and Assessment, 8 December 2017 to 25 January 2018. Geneva: World Health Organization (2018). Available from: http://www.who.int/influenza/human_animal_interface/Influenza_Summary_IRA_HA_interface_25_01_2018_FINAL.pdf?ua=1 (Accessed: March 21, 2018).

[15] Teng Y Bi D Guo X Hu D Feng D Tong Y. Contact reductions from live poultry market closures limit the epidemic of human infections with H7N9 influenza. J Infect (2018) 76:295–394. 10.1016/j.jinf.2017.12.015 29406153

[16] Zaman M Gasimov V Oner AF Dogan N Adisasmito W Coker R Recognizing true H5N1 infections in humans during confirmed outbreaks. J Infect Dev Ctries (2014) 8:202–7. 10.3855/jidc.3329 24518630

[17] Farooqui A Liu W Zeng T Liu Y Zhang L Khan A Probable hospital cluster of H7N9 influenza infection. N Engl J Med (2016) 374(6):596–8. 10.1056/NEJMc1505359 26863372

[18] Imai M Watanabe T Kiso M Nakajima N Yamayoshi S Iwatsuki-Horimoto K A highly pathogenic avian H7N9 influenza virus isolated from a human is lethal in some ferrets infected via respiratory droplets. Cell Host Microbe (2017) 22(5):615–26.e8. 10.1016/j.chom.2017.09.008 29056430 PMC5721358

[19] Lopez-Martinez I Balish A Barrera-Badillo G Jones J Nuñez-García TE Jang Y Highly pathogenic avian influenza A(H7N3) virus in poultry workers, Mexico, 2012. Emerg Infect Dis (2013) 19(9):1531–4. 10.3201/eid1909.130087 23965808 PMC3810917

[20] Watanabe T Kiso M Fukuyama S Nakajima N Imai M Yamada S Characterisation of H7N9 influenza A viruses isolated from humans. Nature (2013) 501(7468):551–5. 10.1038/nature12392 23842494 PMC3891892

[21] Huo X Chen L Qi X Huang H Dai Q Yu H Significantly elevated number of human infections with H7N9 in Jiangsu in eastern China, October 2016 to January 2017. Euro Surveill (2017) 22(13):30496 10.2807/1560-7917.ES.2017.22.13.30496 28382916 PMC5388103

[22] Su S Gu M Liu D Cui J Gao GF Zhou J Epidemiology, evolution, and pathogenesis of H7N9 influenza viruses in five epidemic waves since 2013 in China. Trends Microbiol (2017) 25(9):713–28. 10.1016/j.tim.2017.06.008 28734617

[23] Zhou L Ren R Yang L Bao C Wu J Wang D Sudden increase in human infection with avian influenza A(H7N9) virus in China, September–December 2016. Western Pac Surveill Response J (2017) 8(1):6–14. 10.5365/WPSAR.2017.8.1.001 28409054 PMC5375094

[24] Pinsent A Pepin KM Zhu H Guan Y White MT Riley S The persistance of multiple strains of avian influenza in live bird markets. Proc Biol Sci (2017) 284(1868):20170715 10.1098/rspb.2017.0715 29212718 PMC5740266

[25] Wan XF Dong L Lan Y Long LP Xu C Zou S Indications that live poultry markets are a major source of human H5N1 influenza virus infection in China. J Virol (2011) 85(24):13432–8. 10.1128/JVI.05266-11 21976646 PMC3233185

[26] Bano S Naeem K Malik SA. Evaluation of pathogenic potential of avian influenza virus serotype H9N2 in chickens. Avian Dis (2003) 47(817):817–22. 10.1637/0005-2086-47.s3.817 14575070

[27] Kalaiyarasu S Kumar M Senthil Kumar D Bhatia S Dash SK Bhat S Highly pathogenic avian influenza H5N1 virus induces cytokine dysregulation with suppressed maturation of chicken monocyte-derived dendritic cells. Microbiol Immunol (2016) 60:687–93. 10.1111/1348-0421.12443 27730669

[28] Huang Y Li X Zhang H Chen B Jiang Y Yang L Human infection with an avian influenza A (H9N2) virus in the middle region of China. J Med Virol (2015) 87:1641–8. 10.1002/jmv.24231 25965534

[29] Butt KM Smith GJ Chen H Zhang LJ Leung YH Xu KM Human infection with an avian H9N2 influenza A virus in Hong Kong in 2003. J Clin Microbiol (2005) 43(11):5760–7. 10.1128/JCM.43.11.5760-5767.2005 16272514 PMC1287799

[30] Naguib MM Arafa AS El-Kady MF Selim AA Gunalan V Maurer-Stroh S Evolutionary trajectories and diagnostic challenges of potentially zoonotic avian influenza viruses H5N1 and H9N2 co-circulating in Egypt. Infect Genet Evol (2015) 34:278–91. 10.1016/j.meegid.2015.06.004 26049044

[31] Yu X Jin T Cui Y Pu X Li J Xu J Influenza H7N9 and H9N2 viruses: coexistence in poultry linked to human H7N9 infection and genome characteristics. J Virol (2014) 88(6):3423–31. 10.1128/JVI.02059-13 24403589 PMC3957952

[32] Chen H Yuan H Gao R Zhang J Wang D Xiong Y Clinical and epidemiological characteristics of a fatal case of avian influenza A H10N8 virus infection: a descriptive study. Lancet (2014) 383:714–21. 10.1016/S0140-6736(14)60111-2 24507376

[33] Yuan J Zhang L Kan X Jiang L Yang J Guo Z Origin and molecular characteristics of a novel 2013 avian influenza A(H6N1) virus causing human infection in Taiwan. Clin Infect Dis (2013) 57:1367–8. 10.1093/cid/cit479 23881153

[34] Wei S-H Yang JR Wu HS Chang MC Lin JS Lin CY Human infection with avian influenza A H6N1 virus: an epidemiological analysis. Lancet Respir Med (2013) 1(10):771–8. 10.1016/S2213-2600(13)70221-2 24461756 PMC7164810

[35] Ni F Konrashkina E Wang Q. Structural and functional studies of influenza virus A/H6 hemagglutinin. PLoS One (2015) 10(7):e0134576. 10.1371/journal.pone.0134576 26226046 PMC4520562

[36] Xin L Bai T Zhou JF Chen YK Li XD Zhu WF Seropositivity for avian influenza H6 virus among humans, China. Emerg Infect Dis (2015) 21(7):1267–9. 10.3201/eid2107.150135 26079934 PMC4480397

[37] Hoffmann E Stech J Leneva I Krauss S Scholtissek C Chin PS Characterization of the influenza A virus gene pool in avian species in Southern China: was H6N1 a derivative or a precursor of H5N1? J Virol (2000) 74(14):6309–15. 10.1128/JVI.74.14.6309-6315.2000 10864640 PMC112136

[38] Chin PS Hoffmann E Webby R Webster RG Guan Y Peiris M Molecular evolution of H6 influenza viruses from poultry in Southeastern China: prevalence of H6N1 influenza viruses possessing seven A/Hong Kong/156/97 (H5N1)-like genes in poultry. J Virol (2002) 76(2):507–16. 10.1128/JVI.76.2.507-516.2002 11752141 PMC136834

[39] Lu H Castro AE Pennick K Liu J Yang Q Dunn P Survival of avian influenza virus H7N2 in SPF chickens and their environments. Avian Dis (2003) 47(s3):1015–21. 10.1637/0005-2086-47.s3.1015 14575104

[40] Gharaibeh S Pathogenicity of an avian influenza serotype H9N2 in chickens. Avian Dis (2008) 52(1):106–10. 10.1637/8108-090907-Reg 18459305

[41] França M Stallknecht DE Poulson R Brown J Howerth EW. The pathogenesis of low pathogenic avian influenza in mallards. Avian Dis (2012) 56:976–80. 10.1637/10153-040812-ResNote.1 23402122 PMC11361407

[42] Curran JM Robertson ID Ellis TM Selleck PW O’Dea MA Variation in the responses of wild species of duck, gull, and wader to inoculation with a wild-bird-origin H6N2 low pathogenicity avain influenza virus. Avian Dis (2013) 57(3):581–6. 10.1637/10458-112712-Reg.1 24283122

[43] Wang J Li CC Diao YX Sun XY Hao DM Liu X Different outcomes of infection of chickens and ducks with duck-origin H9N2 influenza A virus. Acta Virol (2014) 58:223–30. 10.4149/av_2014_03_223 25283856

[44] Aly MM Arafa A-SA Hassan MK. Epidemiological findings of outbreaks of disease caused by highly pathogenic H5N1 avian influenza virus in poultry in Egypt during 2006. Avian Dis (2008) 52(2):269–77. 10.1637/8166-103007-Reg.1 18646456

[45] Perkins LEL Swayne DE. Pathobiology of A/Chicken/Hong Kong/220/97 (H5N1) avian influenza virus in seven gallinaceous species. Vet Pathol (2001) 38(2):149–64. 10.1354/vp.38-2-149 11280371

[46] Bingham J Green DJ Lowther S Klippel J Burggraaf S Anderson DE Infection studies with two highly pathogenic avian influenza strains (Vietnamese and Indonesian) in Pekin ducks (*Anas platyrhynchos*), with particular reference to clinical disease, tissue tropism and viral shedding. Avian Pathol (2009) 38(4):267–78. 10.1080/03079450903055371 19937511

[47] Acland HM Silverman Bachin LA Eckroade RJ. Lesions in broiler and layer chickens in an outbreak of highly pathogenic avian influenza virus infection. Vet Pathol (1984) 21:564–9. 10.1177/030098588402100603 6516176

[48] Keawcharoen J van Riel D van Amerongen G Bestebroer T Beyer WE van Lavieren R Wild ducks a long-distance vectors of highly pathogenic avian influenza virus (H5N1). Emerg Infect Dis (2008) 14(4):600–7. 10.3201/eid1404.071016 18394278 PMC2570914

[49] Pantin-Jackwood MJ Swayne DE. Pathobiology of Asian highly pathogenic avian influenza H5N1 virus infections in ducks. Avian Dis (2007) 51(s1):250–9. 10.1637/7710-090606R.1 17494561

[50] Suzuki K Okada H Itoh T Tada T Mase M Nakamura K Association of increased pathogenicity of Asian H5N1 highly pathogenic avian influenza viruses in chickens with highly efficient viral replication accompanied by early destruction of innate immune responses. J Virol (2009) 83(15):7475–86. 10.1128/JVI.01434-08 19457987 PMC2708648

[51] Jeong O-M Kim MC Kim MJ Kang HM Kim HR Kim YJ Experimental infection of chickens, ducks and quails with highly pathogenic H5N1 avian influenza virus. J Vet Sci (2009) 10(1):53–60. 10.4142/jvs.2009.10.1.53 19255524 PMC2801098

[52] Yamamoto Y Nakamura K Yamada M Mase M. Corneal opacity in domestic ducks experimentally infected with H5N1 highly pathogenic avian influenza virus. Vet Pathol (2016) 53(1):65–76. 10.1177/0300985815591077 26123230

[53] Yamamoto Y Nakamura K Yamada M Mase M Comparative pathology of chickens and domestic ducks experimentally infected with highly pathogenic avian influenza viruses (H5N1) isolated in Japan in 2007 and 2008. Jpn Agric Res Q (2010) 44(1):73–80. 10.6090/jarq.44.73

[54] Ito T Couceiro JN Kelm S Baum LG Krauss S Castrucci MR Molecular basis for the generation in pigs of influenza A viruses with pandemic potential. J Virol (1998) 72(9):7367–73. 9696833 10.1128/jvi.72.9.7367-7373.1998 PMC109961

[55] Lipatov AS Kwon YK Sarmento LV Lager KM Spackman E Suarez DL Domestic pigs have low susceptibility to H5N1 highly pathogenic avian influenza viruses. PLoS Pathog (2008) 4(7):e1000102 10.1371/journal.ppat.1000102 18617994 PMC2438613

[56] Zhu Q Yang H Chen W Cao W Zhong G Jiao P A naturally occurring deletion in its NS gene contributes to the attenuation of an H5N1 swine influenza virus in chickens. J Virol (2008) 82(1):220–8. 10.1128/JVI.00978-07 17942562 PMC2224367

[57] Zhou P Hong M Merrill MM He H Sun L Zhang G. Serological report of influenza a (H7N9) infections among pigs in Southern China. BMC Vet Res (2014) 10:203. 10.1186/s12917-014-0203-x 25178684 PMC4236795

[58] Liu Q Zhou B Ma W Bawa B Ma J Wang W Analysis of recombinant H7N9 wild-type and mutant viruses in pigs shows that the Q226L mutation in HA Is important for transmission. J Virol (2014) 88(14):8153–65. 10.1128/JVI.00894-14 24807722 PMC4097782

[59] Balzli C Lager K Vincent A Gauger P Brockmeier S Miller L Susceptibility of swine to H5 and H7 low pathogenic avian influenza viruses. Influenza Other Respi Viruses (2016) 10(4):346–52. 10.1111/irv.12386 PMC4910171 26946338

[60] Yum J Park EH Ku KB Kim JA Oh SK Kim HS Low infectivity of novel avian-origin H7N9 influenza virus in pigs. Arch Virol (2014) 159:2745–9. 10.1007/s00705-014-2143-y 24906526

[61] Jones JC Baranovich T Zaraket H Guan Y Shu Y Webby RJ Human H7N9 influenza A viruses replicate in swine respiratory tissue explants. J Virol (2013) 87(22):12496–8. 10.1128/JVI.02499-13 24027310 PMC3807908

[62] Kwon TY Lee SS Kim CY Shin JY Sunwoo SY Lyoo YS Genetic characterization of H7N2 influenza virus isolated from pigs. Vet Microbiol (2011) 153(3-4):393–7. 10.1016/j.vetmic.2011.06.011 21741185

[63] van Riel D Munster VJ de Wit E Rimmelzwaan GF Fouchier RA Osterhaus AD H5N1 virus attachment to lower respiratory tract. Science (2006) 312(5772):399. 10.1126/science.1125548 16556800

[64] Belser JA Gustin KM Pearce MB Maines TR Zeng H Pappas C Pathogenesis and transmission of avian influenza A (H7N9) virus in ferrets and mice. Nature (2013) 501:556–60. 10.1038/nature12391 23842497 PMC7094885

[65] Zhang Q Shi J Deng G Guo J Zeng X He X H7N9 influenza viruses are transmissible in ferrets by respiratory droplet. Science (2013) 341(6144):410–4. 10.1126/science.1240532 23868922

[66] Herfst S Schrauwen EJ Linster M Chutinimitkul S de Wit E Munster VJ Airborne transmission of influenza A/H5N1 virus between ferrets. Science (2012) 336:1534–41. 10.1126/science.1213362 22723413 PMC4810786

[67] Zaraket H Baranovich T Kaplan BS Carter R Song MS Paulson JC Mammalian adaptation of influenza A(H7N9) virus is limited by a narrow genetic bottleneck. Nat Commun (2015) 6:6553. 10.1038/ncomms7553 25850788 PMC4403340

[68] Edenborough KM Lowther S Laurie K Yamada M Long F Bingham J Predicting disease severity and viral spread of H5N1 influenza virus in ferrets in the context of natural exposure routes. J Virol (2015) 90(4):1888–97. 10.1128/JVI.01878-15 26656692 PMC4733973

[69] Zhu H Wang D Kelvin DJ Li L Zheng Z Yoon SW Infectivity, transmission, and pathology of human-isolated H7N9 influenza virus in ferrets and pigs. Science (2013) 341(6142):183–6. 10.1126/science.1239844 23704376

[70] Tundup S Kandasamy M Perez JT Mena N Steel J Nagy T Endothelial cell tropism is a determinant of H5N1 pathogenesis in mammalian species. PLoS Pathog (2017) 13(3):e1006270. 10.1371/journal.ppat.1006270 28282445 PMC5362246

[71] Gao R Cao B Hu Y Feng Z Wang D Hu W Human infection with a novel avian-origin influenza A (H7N9) virus. N Engl J Med (2013) 368(20):1888–97. 10.1056/NEJMoa1304459 23577628

[72] Wang Z Wan Y Qiu C Quiñones-Parra S Zhu Z Loh L Recovery from severe H7N9 disease is associated with diverse response mechanisms dominated by CD8+ T cells. Nat Commun (2015) 6:6833 10.1038/ncomms7833 25967273 PMC4479016

[73] Hu Y Lu S Song Z Wang W Hao P Li J Association between adverse clinical outcome in human disease caused by novel influenza A H7N9 virus and sustained viral shedding and emergence of antiviral resistance. Lancet (2013) 381(9885):2273–9. 10.1016/S0140-6736(13)61125-3 23726392

[74] Zhang Z Zhang J Huang K Li KS Yuen KY Guan Y Systemic infection of avian influenza A virus H5N1 subtype in humans. Hum Pathol (2009) 40:735–9. 10.1016/j.humpath.2008.08.015 19121843 PMC7112124

[75] To KF Chan PK Chan KF Lee WK Lam WY Wong KF Pathology of fatal human infection associated with avian influenza A H5N1 virus. J Med Virol (2001) 63:242–6. 10.1002/1096-9071(200103)63:3<242::AID-JMV1007>3.0.CO;2-N 11170064

[76] Kuchipudi SV Tellabati M Sebastian S Londt BZ Jansen C Vervelde L Highly pathogenic avian influenza virus infection in chickens but not ducks is associated with elevated host immune and pro-inflammatory responses. Vet Res (2014) 45:118 10.1186/s13567-014-0118-3 25431115 PMC4246556

[77] Short KR Veeris R Leijten LM van den Brand JM Jong VL Stittelaar K Proinflammatory cytokine responses in extra-respiratory tissues during severe influenza. J Infect Dis (2017) 216:829–33. 10.1093/infdis/jix281 28973159

[78] Shinya K Gao Y Cilloniz C Suzuki Y Fujie M Deng G Integrated clinical, pathologic, virologic, and transcriptomic analysis of H5N1 influenza virus-induced viral pneumonia in the rhesus macaque. J Virol (2012) 86(11):6055–66. 10.1128/JVI.00365-12 22491448 PMC3372212

[79] Gao S Kang Y Yuan R Ma H Xiang B Wang Z Immune responses of chickens infected with wild bird-origin H5N6 avian influenza virus. Front Microbiol (2017) 8:1081. 10.3389/fmicb.2017.01081 28676793 PMC5476689

[80] Meliopoulos VA Karlsson EA Kercher L Cline T Freiden P Duan S Human H7N9 and H5N1 influenza viruses differ in induction of cytokines and tissue tropism. J Virol (2014) 88(22):12982–91. 10.1128/JVI.01571-14 25210188 PMC4249090

[81] To KK Lau CC Woo PC Lau SK Chan JF Chan KH Human H7N9 virus induces a more pronounced pro-inflammatory cytokine but an attenuated interferon response in human bronchial epithelial cells when compared with an epidemiologically-linked chicken H7N9 virus. Virol J (2016) 13:42. 10.1186/s12985-016-0498-2 26975414 PMC4791762

[82] Wu W Shi D Fang D Guo F Guo J Huang F A new perspective on C-reactive protein in H7N9 infections. Int J Infect Dis (2016) 44:31–6. 10.1016/j.ijid.2016.01.009 26809124

[83] Zhao C Qi X Ding M Sun X Zhou Z Zhang S Pro-inflammatory cytokine dysregulation is associated with novel avian influenza A (H7N9) virus in primary human macrophages. J Gen Virol (2016) 97(2):299–305. 10.1099/jgv.0.000357 26644088

[84] Nelli RK Dunham SP Kuchipudi SV White GA Baquero-Perez B Chang P Mammalian innate resistance to highly pathogenic avian influenza H5N1 virus infection is mediated through reduced proinflammation and infectious virus release. J Virol (2012) 86(17):9201–10. 10.1128/JVI.00244-12 22718824 PMC3416141

[85] Turner MD Nedjai B Hurst T Pennington DJ. Cytokines and chemokines: at the crossroads of cell signalling and inflammatory disease. Biochim Biophys Acta (2014) 1843:2563–82. 10.1016/j.bbamcr.2014.05.014 24892271

[86] Boonnak K Vogel L Feldmann F Feldmann H Legge KL Subbarao K Lymphopenia associated with highly virulent H5N1 virus infection due to plasmocytoid dendritic cell mediated apoptosis of T cells. J Immunol (2014) 192(12):5906–12. 10.4049/jimmunol.1302992 24829418 PMC4083746

[87] Waring P Müllbacher A Cell death induced by the Fas/Fas ligand pathway and its role in pathology. Immunol Cell Biol (1999) 77:312–7. 10.1046/j.1440-1711.1999.00837.x 10457197

[88] Chen Y Liang W Yang S Wu N Gao H Sheng J Human infections with the emerging avian influenza A H7N9 virus from wet market poultry: clinical analysis and characterisation of viral genome. Lancet (2013) 381:1916–25. 10.1016/S0140-6736(13)60903-4 23623390 PMC7134567

[89] Uiprasertkul M Kitphati R Puthavathana P Kriwong R Kongchanagul A Ungchusak K Apoptosis and pathogenesis of avian influenza A (H5N1) virus in humans. Emerg Infect Dis (2007) 13(5):708–12. 10.3201/eid1305.060572 17553248 PMC2738443

[90] Wang C Yu H Horby PW Cao B Wu P Yang S Comparison of patients hospitalized with influenza A subtypes H7N9, H5N1, and 2009 pandemic H1N1. Clin Infect Dis (2014) 58(8):1095–103. 10.1093/cid/ciu053 24488975 PMC3967826

[91] Wang Z Zhu L Nguyen THO Wan Y Sant S Quiñones-Parra SM Clonally diverse CD38+HLA-DR+CD8+ T cells persist during fatal H7N9. Nat Commun (2018) 9:824 10.1038/s41467-018-03243-7 29483513 PMC5827521

[92] Nichols JE Niles JA Roberts NJ Jr. Human lymphocyte apoptosis after exposure to influenza A virus. J Virol (2001) 73(13):5921–9. 10.1128/JVI.73.13.5921-5929.2001 11390593 PMC114307

[93] Herfst S Imai M Kawaoka Y Fouchier RA. Avian influenza virus transmission to mammals. Curr Top Microbiol Immunol (2014) 385:137–55. 10.1007/82_2014_387 25048542

[94] Rogers GN Pritchett TJ Lane JL Paulson JC. Differential sensitivity of human, avian, and equine influenza A viruses to a glycoprotein inhibitor of infection: selection of receptor specific variants. Virology (1983) 131(2):394–408. 10.1016/0042-6822(83)90507-X 6197808

[95] de Vries RP Peng W Grant OC Thompson AJ Zhu X Bouwman KM Three mutations switch H7N9 influenza to human-type receptor specificity. PLoS Pathog (2017) 13(6):e1006390. 10.1371/journal.ppat.1006390 28617868 PMC5472306

[96] Dortmans JC Dekkers J Wickramasinghe IN Verheije MH Rottier PJ van Kuppeveld FJ Adaptation of novel H7N9 influenza virus to human receptors. Sci Rep (2013) 3:3058 10.1038/srep03058 24162312 PMC3808826

[97] Chandrasekaran A Srinivasan A Raman R Viswanathan K Raguram S Tumpey TM Glycan topology determines human adaptation of avian H5N1 virus hemagglutinin. Nat Biotechnol (2008) 26(1):107–13. 10.1038/nbt1375 18176555

[98] Chrzastek K Lee DH Gharaibeh S Zsak A Kapczynski DR. Characterization of H9N2 avian influenza viruses from the Middle East demonstrates heterogeneity at amino acid position 226 in the hemagglutinin and potential for transmission to mammals. Virology (2018) 518:195–201. 10.1016/j.virol.2018.02.016 29524835

[99] Li X Shi J Guo J Deng G Zhang Q Wang J Genetics, receptor binding property, and transmissibility in mammals of naturally isolated H9N2 avian influenza viruses. PLoS Pathog (2014) 10(11):e1004508. 10.1371/journal.ppat.1004508 25411973 PMC4239090

[100] Watanabe Y Arai Y Daidoji T Kawashita N Ibrahim MS El-Gendy E-D Characterization of H5N1 influenza virus variants with hemagglutinin mutations isolated from patients. MBio (2015) 6(2):e00081-15 10.1128/mBio.00081-15 25852160 PMC4453573

[101] Watanabe Y Ibrahim MS Ellakany HF Kawashita N Mizuike R Hiramatsu H Acquisition of human-type receptor binding specificity by new H5N1 influenza virus sublineages during their emergence in birds in Egypt. PLoS Pathog (2011) 7(5):e1002068. 10.1371/journal.ppat.1002068 21637809 PMC3102706

[102] Steel J Lowen AC Mubareka S Palese P. Transmission of influenza virus in a mammalian host is increased by PB2 amino acids 627K or 627E/701N. PLoS Pathog (2009) 5(1):e1000252. 10.1371/journal.ppat.1000252 19119420 PMC2603332

[103] Labadie K Dos Santos Afonso E Rameix-Welti MA van der Werf S Naffakh N Host-range determinants on the PB2 protein of influenza A viruses control the interaction between the viral polymerase and nucleoprotein in human cells. Virology (2007) 362:271–82. 10.1016/j.virol.2006.12.027 17270230

[104] Yu K Liu X Qi T Yang H Whitfield DM Y Chen Q General low-temperature reaction pathway from precursors to monomers before nucleation of compound semiconductor nanocrystals. Nat Commun (2016) 7:12223. 10.1038/ncomms12223 27531507 PMC4992053

[105] Zhang H Li X Guo J Li L Chang C Li Y The PB2 E627K mutation contributes to the high polymerase activity and enhanced replication of H7N9 influenza virus. J Gen Virol (2014) 95:779–86. 10.1099/vir.0.061721-0 24394699

[106] Schrauwen EJ Herfst S Leijten LM van Run P Bestebroer TM Linster M The multibasic cleavage site in H5N1 virus is critical for systemic spread along the olfactory and hematogenous routes in ferrets. J Virol (2012) 86(7):3975–84. 10.1128/JVI.06828-11 22278228 PMC3302532

[107] Suguitan AL Matsuoka Y Lau YF Santos CP Vogel L Cheng LI The multibasic cleavage site of hemagglutinin of highly pathogenic A/Vietnam/1203/2004 (H5N1) avian influenza virus acts as a virulence factor in a host-specific manner in mammals. J Virol (2012) 86(5):2706–14. 10.1128/JVI.05546-11 22205751 PMC3302284

[108] Stieneke-Gröber A Vey M Angliker H Shaw E Thomas G Roberts C Influenza virus hemagglutinin with a multibasic cleavage site is activated by furin, a subtilisin-like endoprotease. EMBO J (1992) 11(7):2407–14. 1628614 10.1002/j.1460-2075.1992.tb05305.x PMC556715

[109] Hagag IT Mansour SM Zhang Z Ali AA Ismaiel el-BM Salama AA Pathogenicty of highly pathogenic avian influenza virus H5N1 in naturally infected poultry in Egypt. PLoS One (2015) 10(5):e0120061 10.1371/journal.pone.0120061 25962145 PMC4427178

[110] Berhane Y Hisanaga T Kehler H Neufeld J Manning L Argue C Highly pathogenic avian influenza virus A (H7N3) in domestic poultry, Saskatchewan, Canada, 2007. Emerg Infect Dis (2009) 15(9):1492–5. 10.3201/eid1509.080231 19788823 PMC2819867

[111] Killian ML Kim-Torchetti M Hines N Yingst S DeLiberto T Lee DH. Outbreak of H7N8 low pathogenic avian influenza in commercial Turkeys with spontaneous mutation to highly pathogenic avian influenza. Genome Announc (2016) 4(3):e00457-16. 10.1128/genomeA.00457-16 27313288 PMC4911467

[112] Stech O Veits J Weber S Deckers D Schröer D Vahlenkamp TW Acquisition of a polybasic hemagglutinin cleavage site by a low-pathogenic avian influenza virus is not sufficient for immediate transformation into a highly pathogenic strain. J Virol (2009) 83(11):5864–8. 10.1128/JVI.02649-08 19297482 PMC2681970

[113] Wang QM Liu SL Chen EF [Advances on epidemiological research of human infections with novel avian influenza A (H7N9) virus]. Zhonghua Yu Fang Yi Xue Za Zhi (2017) 51(2):183–7. 10.3760/cma.j.issn.0253-9624.2017.02.017 28219161

[114] Imai M Watanabe T Hatta M Das SC Ozawa M Shinya K Experimental adaptation of an influenza H5 haemagglutinin (HA) confers respiratory droplet transmission to a reassortant H5 HA/H1N1 virus in ferrets. Nature (2012) 486:420–8. 10.1038/nature10831 22722205 PMC3388103

[115] Haller O Gao S von der Malsburg A Daumke O Kochs G. Dynamin-like MxA GTPase: structural insights into oligomerization and implications for antiviral activity. J Biol Chem (2010) 285(37):28419–24. 10.1074/jbc.R110.145839 20538602 PMC2937866

[116] Zimmermann P Mänz B Haller O Schwemmle M Kochs G. The viral nucleoprotein determines Mx sensitivity of influenza A viruses. J Virol (2011) 85(16):8133–40. 10.1128/JVI.00712-11 21680506 PMC3147989

[117] Mänz B Dornfeld D Götz V Zell R Zimmermann P Haller O Pandemic influenza A viruses escape from restriction by human MxA though adaptive mutations in the nucleoprotein. PLoS Pathog (2013) 9(3):e1003279 10.1371/journal.ppat.1003279 23555271 PMC3610643

[118] Deeg CM Hassan E Mutz P Rheinemann L Götz V Magar L In vivo evasion of MxA by avian influenza viruses requires human signature in the viral nucleoprotein. J Exp Med (2017) 214(5):1239–48. 10.1084/jem.20161033 28396461 PMC5413327

[119] Schusser B Reuter A von der Malsburg A Penski N Weigend S Kaspers B Mx Is dispensable for interferon-mediated resistance of chicken cells against influenza A virus. J Virol (2011) 85(16):8307–15. 10.1128/JVI.00535-11 21632756 PMC3147972

[120] Benfield CTO Lyall JW Tiley LS. The cytoplasmic location of chicken Mx is not the determining factor for its lack of antiviral activity. PLoS One (2010) 5(8):e12151. 10.1371/journal.pone.0012151 20808435 PMC2922328

[121] Qi X Zhang H Wang Q Wang J. The NS1 protein of avian influenza virus H9N2 induces oxidative-stress-mediated chicken oviduct epithelial cells apoptosis. J Gen Virol (2016) 97:3183–92. 10.1099/jgv.0.000625 27902334

[122] Burggraaf S Bingham J Payne J Kimpton WG Lowenthal JW Bean AG Increased inducible nitric oxide synthase expression in organs is associated with a higher severity of H5N1 influenza infection. PLoS One (2011) 6(1):e14561 10.1371/journal.pone.0014561 21283521 PMC3023712

[123] Jia D Influenza virus non-structural protein 1 (NS1) disrupts interferon signalling. PLoS One (2010) 5(11):e13927 10.1371/journal.pone.0013927 21085662 PMC2978095

[124] Wang J Zeng Y Xu S Yang J Wang W Zhong B A naturally occurring deletion in the effector domain of H5N1 swine influenza virus nonstructural protein 1 regulates viral fitness and host innate immunity. J Virol (2018) 92(11):e00149-18 10.1128/JVI.00149-18 29563291 PMC5952131

[125] Jiao P Tian G Li Y Deng G Jiang Y Liu C A single-amino-acid substitution in the NS1 protein changes the pathogenicity of H5N1 avian influenza viruses in mice. J Virol (2008) 82(3):1146–54. 10.1128/JVI.01698-07 18032512 PMC2224464

[126] Wang L Fu X Zheng Y Zhou P Fang B Huang S The NS1 protein of H5N6 feline influenza virus inhibits feline beta interfereon response by preventing NF-κB and IRF3 activation. Dev Comp Immunol (2017) 74:60–8. 10.1016/j.dci.2017.04.003 28395999 PMC7173090

[127] Thube MM Shil P Kasbe R Patil AA Pawar SD Mullick J. Differences in Type I interferon response in human lung epithelial cells infected by highly pathogenic H5N1 and low pathogenic H11N1 avian influenza viruses. Virus Genes (2018) 54(3):414–23. 10.1007/s11262-018-1556-1 29574656

[128] Ayllon J Domingues P Rajsbaum R Miorin L Schmolke M Hale BG A single amino acid substitution in the novel H7N9 influenza A NS1 protein increases CPSF30 binding and virulence. J Virol (2014) 88(20):12146–51. 10.1128/JVI.01567-14 25078692 PMC4178744

[129] Bailey CC Kondur HR Huang IC Farzan M. Interferon-induced transmembrane protein 3 is a type II transmembrane protein. J Biol Chem (2013) 288(45):32184–93. 10.1074/jbc.M113.514356 24067232 PMC3820858

[130] Bailey CC Zhong G Huang IC Farzan M. IFITM-family proteins: the cell’s first line of antiviral defense. Annu Rev Virol (2014) 1:261–83. 10.1146/annurev-virology-031413-085537 25599080 PMC4295558

[131] Feeley EM Sims JS John SP Chin CR Pertel T Chen LM IFITM3 inhibits influenza A virus infection by preventing cytosolic entry. PLoS Pathog (2011) 7(10):e1002337. 10.1371/journal.ppat.1002337 22046135 PMC3203188

[132] Brass AL Huang IC Benita Y John SP Krishnan MN Feeley EM The IFITM proteins mediate cellular resistance to influenza A H1N1 virus, West Nile virus, and dengue virus. Cell (2009) 139:1243–54. 10.1016/j.cell.2009.12.017 20064371 PMC2824905

[133] Jia R Pan Q Ding S Rong L Liu SL Geng Y The N-terminal region of IFITM3 modulates its antiviral activity by regulating IFITM3 cellular localization. J Virol (2012) 86(24):13697–707. 10.1128/JVI.01828-12 23055554 PMC3503121

[134] Wakim LM Gupta N Mintern JD Villadangos JA Enhanced survival of tissue-resident memory CD8+ T cells during infection with influenza virus due to selective expression of IFITM3. Nat Immunol (2013) 14(3):238–46. 10.1038/ni.2525 23354485

[135] Yang X Tan B Zhou X Xue J Zhang X Wang P Interferon-inducible transmembrane protein 3 genetic variant rs12252 and influenza susceptibility and severity: a meta-analysis. PLoS One (2015) 10(5):e0124985. 10.1371/journal.pone.0124985 25942469 PMC4420464

[136] Zhang YH Zhao Y Li N Peng YC Giannoulatou E Jin RH Interferon-induced transmembrane protein-3 genetic variant rs12252-C is associated with severe influenza in Chinese individuals. Nat Commun (2013) 4:1418. 10.1038/ncomms2433 23361009 PMC3562464

[137] Wang Z Zhang A Wan Y Liu X Qiu C Xi X Early hypercytokinemia is associated with interferon-induced transmembrane protein-3 dysfunction and predictive of fatal H7N9 infection. Proc Natl Acad Sci U S A (2014) 111(2):769–74. 10.1073/pnas.1321748111 24367104 PMC3896201

[138] Williams DE Wu WL Grotefend CR Radic V Chung C Chung YH IFITM3 polymorphism rs12252-C restricts influenza A viruses. PLoS One (2014) 9(10):e110096. 10.1371/journal.pone.0110096 25314048 PMC4196997

[139] Makvandi-Nejad S Laurenson-Schafer H Wang L Wellington D Zhao Y Jin B Lack of truncated IFITM3 transcripts in cells homozygous for the rs12252-C variant that is associated with severe influenza infection. J Infect Dis (2018) 217:257–62. 10.1093/infdis/jix512 29202190

[140] Allen EK Randolph AG Bhangale T Dogra P Ohlson M Oshansky CM SNP-mediated disruption of CTCF binding at the IFITM3 promoter is associated with risk of severe influenza in humans. Nat Med (2017) 23:975–83. 10.1038/nm.4370 28714988 PMC5702558

[141] Yu M Qi W Huang Z Zhang K Ye J Liu R Expression profile and histological distribution of IFITM1 and IFITM3 during H9N2 avian influenza virus infection in BALB/c mice. Med Microbiol Immunol (2015) 204:505–14. 10.1007/s00430-014-0361-2 25265877 PMC7087031

[142] Benfield CTO Smith SE Wright E Wash RS Ferrara F Temperton NJ Bat and pig IFN-induced transmembrane protein 3 restrict cell entry by influenza virus and lyssavirus. J Gen Virol (2015) 96:991–1005. 10.1099/vir.0.000058 25614588 PMC4631062

[143] Smith SE Gibson MS Wash RS Ferrara F Wright E Temperton N Chicken interferon-inducible transmembrane protein 3 restricts influenza viruses and lyssaviruses in vitro. J Virol (2013) 87(23):12957–66. 10.1128/JVI.01443-13 24067955 PMC3838109

[144] Cao Y Huang Y Xu K Liu Y Li X Xu Y Differential responses of innate immunity triggered by different subtypes of influenza a viruses in human and avian hosts. BMC Med Genomics (2017) 10:70. 10.1186/s12920-017-0304-z 29322931 PMC5763291

[145] Smith J Smith N Yu L Paton IR Gutowska MW Forrest HL A comparative analysis of host responses to avian influenza infection in ducks and chickens highlights a role for the interferon-induced transmembrane proteins in viral resistance. BMC Genomics (2015) 16:574. 10.1186/s12864-015-1778-8 26238195 PMC4523026

[146] Cheng VC Chan JF Wen X Wu WL Que TL Chen H Infection of immunocompromised patients by avian H9N2 influenza A virus. J Infect (2011) 62:394–9. 10.1016/j.jinf.2011.02.007 21356238

[147] Law AH Lee DC Yuen KY Peiris M Lau AS. Cellular response to influenza virus infection: a potential role for autophagy in CXCL10 and interferon-alpha induction. Cell Mol Immunol (2010) 7:263–70. 10.1038/cmi.2010.25 20473322 PMC4003230

[148] Nili H Asasi K. Avian influenza (H9N2) outbreak in Iran. Avian Dis (2003) 47(s3):828–31. 10.1637/0005-2086-47.s3.828 14575072

[149] Nili H Asasi K. Natural cases and an experimental study of H9N2 avian influenza in commercial broiler chickens of Iran. Avian Pathol (2002) 31(3):247–52. 10.1080/03079450220136567 12396348

[150] Simonsen L Spreeuwenberg P Lustig R Taylor RJ Fleming DM Kroneman M Global mortality estimates for the 2009 influenza pandemic from the GLaMOR project: a modeling study. PLoS Med (2013) 10(11):e1001558. 10.1371/journal.pmed.1001558 24302890 PMC3841239

[151] Huang SS Banner D Degousee N Leon AJ Xu L Paquette SG Differential pathological and immune responses in newly weaned ferrets are associated with a mild clinical outcome of pandemic 2009 H1N1 infection. J Virol (2012) 86(24):13187–201. 10.1128/JVI.01456-12 23055557 PMC3503035

[152] Helferty M Vachon J Tarasuk J Rodin R Spika J Pelletier L. Incidence of hospital admissions and severe outcomes during the first and second waves of pandemic (H1N1) 2009. CMAJ (2010) 182(18):1981–7. 10.1503/cmaj.100746 21059773 PMC3001504

[153] Paquette SG Huang SSH Banner D Xu L Leόn A Kelvin AA Impaired heterologous immunity in aged ferrets during sequential influenza A H1N1 infection. Virology (2014) 46(4–465):177–83. 10.1016/j.virol.2014.07.013 PMC4157083 25086242

[154] Zhou L Tan Y Kang M Liu F Ren R Wang Y Preliminary epidemiology of human infections with highly pathogenic avian influenza A(H7N9) virus, China, 2017. Emerg Infect Dis (2017) 23(8):1355–9. 10.3201/eid2308.170640 28580900 PMC5547798

[155] Mackay IM Influenza A(H7N9) Virus: Detection Numbers and Graphs…. VDU’s blog (2014).

[156] Gostic KM Ambrose M Worobey M Lloyd-Smith JO. Potent protection against H5N1 and H7N9 influenza via childhood hemagglutinin imprinting. Science (2016) 354(6313):722–6. 10.1126/science.aag1322 27846599 PMC5134739

[157] Valkenburg SA Josephs TM Clemens EB Grant EJ Nguyen TH Wang GC Molecular basis for universal HLA-A*0201-restricted CD8+ T-cell immunity against influenza viruses. Proc Natl Acad Sci U S A (2016) 113(16):4440–5. 10.1073/pnas.1603106113 27036003 PMC4843436

[158] Quiñones-Parra S Grant E Loh L Nguyen TH Campbell KA Tong SY Preexisting CD8+ T-cell immunity to the H7N9 influenza A virus varies across ethnicities. Proc Natl Acad Sci U S A (2014) 111(3):1049–54. 10.1073/pnas.1322229111 24395804 PMC3903243

[159] Nguyen THO Sant S Bird NL Grant EJ Clemens EB Koutsakos M Perturbed CD8+ T cell immunity across universal influenza epitopes in the elderly. J Leukoc Biol (2017) 103(2):321–39. 10.1189/jlb.5MA0517-207R 28928269

[160] Clemens EB Grant EJ Wang Z Gras S Tipping P Rossjohn J Towards identification of immune and genetic correlates of severe influenza disease in Indigenous Australians. Immunol Cell Biol (2016) 94:367–77. 10.1038/icb.2015.93 26493179 PMC4840236

[161] Hertz T Oshansky CM Roddam PL DeVincenzo JP Caniza MA Jojic N HLA targeting efficiency correlates with human T-cell response magnitude and with mortality from influenza A infection. Proc Natl Acad Sci U S A (2013) 110(33):13492–7. 10.1073/pnas.1221555110 23878211 PMC3746844

[162] Liu R Moise L Tassone R Gutierrez AH Terry FE Sangare K H7N9 T-cell epitopes that mimic human sequences are less immunogenic and may induce Treg-mediated tolerance. Hum Vaccin Immunother (2015) 11(9):2241–52. 10.1080/21645515.2015.1052197 26090577 PMC4635734

[163] Hou Y Guo Y Wu C Shen N Jiang Y Wang J. Prediction and identification of T cell epitopes in the H5N1 influenza virus nucleoprotein in chicken. PLoS One (2012) 7(6):e39344. 10.1371/journal.pone.0039344 22745738 PMC3379973

[164] Zhang W Huang Q Lu M Zhu F Huang YY Yang SH Exploration of the BF2*15 major histocompatibility complex class I binding motif and identification of cytotoxic T lymphocyte epitopes from the H5N1 influenza virus nucleoprotein in chickens. Arch Virol (2016) 161:3081–93. 10.1007/s00705-016-3013-6 27518404

[165] Fleming-Canepa X Jensen SM Mesa CM Diaz-Satizabal L Roth AJ Parks-Dely JA Extensive allelic diversity of MHC class I in wild mallard ducks. J Immunol (2016) 197:783–94. 10.4049/jimmunol.1502450 27342841

[166] Wu Y Wang J Fan S Chen R Liu Y Zhang J Structural definition of duck major histocompatibility complex class I molecules that might explain efficient cytotoxic T lymphocyte immunity to influenza A virus. J Virol (2017) 91(14):e02511-16. 10.1128/JVI.02511-16 28490583 PMC5487541

[167] Buehler DM Verkuil YI Tavares ES Baker AJ. Characterization of MHC class I in a long-distance migrant shorebird suggests multiple transcribed genes and intergenic recombination. Immunogenetics (2013) 65:211–25. 10.1007/s00251-012-0669-2 23239370

[168] Watanabe T Zhong G Russell CA Nakajima N Hatta M Hanson A Circulating avian influenza viruses closely related to the 1918 virus have pandemic potential. Cell Host Microbe (2014) 15:692–705. 10.1016/j.chom.2014.05.006 24922572 PMC4205238

